# Diversity and bioprospecting for industrial hydrolytic enzymes of microbial communities isolated from deserted areas of south-east Morocco

**DOI:** 10.3934/microbiol.2022002

**Published:** 2022-01-20

**Authors:** Amina Manni, Abdelkarim Filali-Maltouf

**Affiliations:** Department of Biology, Mohammed V University, Laboratory of microbiology and molecular biology, Mohammed V university, *Rabat*, Av Ibn Batouta BP 1014, Morocco

**Keywords:** bacterial diversity, desert, enzyme activity, stress tolerance, Sahara

## Abstract

The current study aimed to analyze bacterial communities' diversity and abundance in three different deserted areas (Merzouga, Mhamid Elghizlane, and Erg lihoud) located in Moroccan Sahara, as well as to investigate osmotolerant microorganisms producing hydrolytic enzymes. The isolates were taxonomically affiliated using 16S rRNA gene sequencing. Four different hydrolase activities (amylase, lipase, cellulase, and protease) and osmotic stress tolerance were evaluated. The phylogenetic analysis of 364 screened isolates belonged to three phyla (Firmicutes 73%, Proteobacteria 26% and Actinobacteria 1%) and 18 different genera, from *Bacillus, Ornithinibacillus, Paenibacillus, Geobacillus, Pseudomonas, Acinetobacter, Agrobacterium, Arthrobacter, Paenarthrobacter, Enterobacter, Staphylococcus, Erwinia, Herbasprillum, Ocuria, Massilia, Planomicrobium, Hodococcus, and Stenotrophomonas*. The results detected a high proportion of osmotolerant and enzymes producing bacteria, many isolates can tolerate up to 55 °C (40%, 28%, and 30% in Merzouga, Mhamid Elghizlane, and Erg lihoudi, respectively). Meanwhile, the salinity tolerance reached 12% in some isolates with different proportions in each site, 29% in Merzouga, 24% in Mhamid Elghizlane, and 9% in Erg lihoudi. Furthermore, the enzymatic tests showed the presence of an amylolytic, lipolytic, cellulolytic, proteolytic activities in 20%, 31%, 63% and 72% of total strains, respectively.

As a result, the present study is thus a preliminary yet critical step towards identifying the best bacterial candidates for further biotechnological applications.

## 1. Introduction

Our planet contains a large number of challenging environments like desert who are known to be exposed ecosystems to prolonged moisture deficit periods representing the driest places on Earth. Also known as arid-deserts, they cover nearly 33% of the Earth's land surface comprising the largest land surface area [1]. With increasing global desertification due to the current global warming trends drylands are still in amplification [2]. By the end of the century, their expansion will attend more than half of land surfaces [3]. Which make them play a substantial part in the biogeochemical cycles of numerous chemical elements and have an impact on the gas emission in the atmosphere [4],[5]. Many of them are distributed worldwide and categorized in four main types, polar frost, polar tundra, cold and hot deserts [6]. They represent harsh environments that are characterized by several limiting factors such as water scarcity, high ultraviolet radiation, soil alkalinity and salinity, extreme temperatures fluctuations and nutrient poor availability [7],[8]. Moreover, increased aridity in global drylands diminishes microbial soil diversity and abundance [9]. Due to desert special characteristics some of them are considered great analog areas and primers for astrobiological investigations [10],[11].

Soil biotic composition and activity are highly influenced by the presence, the distribution and the variation in chemical composition of organic matter [12]. In arid and semiarid ecosystems, soil biotic functions are mostly modulated by the interaction between organic matter availability and moisture [13]. To cope with those harsh conditions, soil microorganism developed survival strategies by making changes in the composition of the cell envelope. These changes can be manifested through the formation of biofilms and endospores, the production of general shock proteins and chaperones or the expression of transcriptional regulators [14]. Recently, two more other survival strategies hypothesis sustaining dormancy in arid ecosystems have been reported; the continual-energy-harvesting hypothesis and the energy reserve hypothesis which depend on the severity of different environmental parameters as extensively explained by Leung et al. [15]. Consequently, both of hot and cold deserts harbor a high bacterial diversity [16],[17]. For a long time, these bacteria were thought to be environmental changes predictors [18].

Microbial communities living in these environments cease to metabolize complex organic substrates that higher organisms cannot degrade [19], which make them able to produce high amounts of enzyme substances that have potential applications in a broad range of industrial, agricultural and medical processes [20],[21]. These environments have provided a useful source of novel active enzymes from several microorganisms endowing special abilities; halophiles, thermophiles, acidophiles, alkaliphiles, and haloalkaliphiles, etc.[22],[23]. Microorganism-derived enzymes provide a number of benefits, including low cost, high stability and substrate solubility, increased product recovery, high mass transfer rate, regular availability and better-quality [24]. Currently, many research organizations expect that Industrial enzymes will hit $8.7 billion by 2026 with an annual growth rate of 6, 3% [25],[26]. 75% of them are hydrolytic enzymes [27] and most thermostable enzymes studied are protease, amylase, cellulase and lipase [28]. As a result, worldwide an increasing number of researchers have advocated identifying viable functional strains from a variety of harsh habitats due to their ability to harbor hot active enzymes producing bacteria. In morocco, hot deserts such us Moroccan Sahara desert remain an insufficiently explored area. Till now, only few studies limited to one station (Merzouga) in the Moroccan Sahara desert have described microbial communities. Therefore, understanding the extent of bacterial diversity still incomplete. Efforts are still being made to obtain a comprehensive view of microbial community composition and structure, in desert environments.

The overall goal of the current research is to attempted a better understanding of the microbial community diversity in Moroccan Sahara desert, relying on phylogenetic analysis of rRNA gene (16S rDNA) sequences [29] in three different regions which two of them are studied for the first time. Further, the isolates osmotic stress tolerance and hydrolytic enzymes activities were explored in order to create a resourceful enzyme repertoire in bacterial desert community promoting large-scale applications in the biotechnological field.

## 2. Materials and methods

### 2.1. Sampling sites and bacteria isolation

Bacterial strains were isolated from non-vegetated sand dunes covering three different regions of Moroccan Sahara desert. Samples were collected in three replicates from the following geometric points: (X = −3, 97852083325, Y = 31, 1093333325), (X = −5, 722877, Y = 29, 831673), (X = −5, 686705, Y = 29, 90211), representing Merzouga, Mhamid Elghizlane and Erg lihoudi, respectively. The samples, from 0–5 cm depth, were immediately transported to the laboratory. From each sample, 1g of samples was suspended in 9 ml physiologic water and shaken vigorously for 30 min. Serial dilutions (from 10–3 to 10–7) were made. Then 0.1 mL from each dilutions was spread in an appropriate agar based media. Bacterial samples were stored in 15% glycerol at −80 °C.

### 2.2. Physio-chemical and climatic parameters

Physico-chemical properties analysis and ombrothermic parameters were proceeded in order to test whether the isolated bacteria are distributed according to abiotic or to spacial factors. To this end, soil samples were pooled and analyzed according to standard quality control procedures (SSSA, 1996) at IAV (Agronomic and veterinary institute Hassan II, pedoloy laboratory, Morocco) and ombrothermic parameters were analysed by CHIRPS [30].

### 2.3. Molecular analysis

Bacterial pellets were suspended in extraction buffer (100 mM Tris–HCl pH = 8, 100 mM EDTA pH = 8, 5% SDS, NaCl and RNAase), mixed and incubated 5-10 min in ice. The supernatant fluid was collected after a 10 min centrifugation at 13,000g at room temperature. The nucleic acids were extracted by the addition of an equal volume of chloroform/isoamyl alcohol (24:1) followed by centrifugation at 13,000 g for 5 min. DNA was precipitated by addition of double volume of 100% ethanol, centrifuged and washed by 70% ethanol. The DNA pellets were then airdried and resuspended in 50 µL 1/10 TE buffer (1 mM Tris-HCl pH 8, 0.1 mM Na EDTA pH 8).

Recovered DNA was quantified using NanoDrop™ (Thermo Scientific, Waltham, MA, USA). The 16S rDNA was amplified using the universal primers FD1: 5′CCGAATTCGTCGACAACAGAGTTTGATCCTGGCTCAG-3′ and RS16: 5′-TACGGCTACCTTGTTACGACTT-3′. The 25-µL PCR mixture contained 20 ng bacterial DNA, 250 pmol each primer, 5 µL 10X PCR buffer, 2.5 U Taq DNA polymerase (Bioline, Morocco). The PCR temperature cycling program consisted of an initial denaturation at 94 °C for 5 min then 35 cycles of three levels 94 °C for 1 min, 56 °C for 1 min, and 72°C for 1 min. Ultimately, by a final extension at 72 °C was applied for 7 min. The PCR products were examined by running them on a 1% agarose gel, and the desirePCR product was purified using the PCR Purification Kit (Promega, USA). The PCR products sequencing were performed by Genoscreen using 3730xl DNA Sanger sequencer at Pasteur institute (Lille, France).

### 2.4. Bioinformatic and statistical analyses

The sequences obtained were initially compared with reference sequences by using BLAST available in the National Centre for Biotechnology Information (NCBI). The 16S rRNA gene sequences were aligned using the multiple alignment program Genedoc 2.7 software. The phylogenetic tree construction was carried out in The Molecular Evolutionary Genetics Analysis (MEGA-X software) [31].The sequences of the 16S rDNA gene were submitted to the GenBank database under accession numbers from KX013406 to KX013441. In order to compare the bacterial diversity within the three sites, the 16S rRNA gene sequences were used to analyze diversity index: Evenness (J) and the Simpson's index (D). To reveal bacterial communities similarity between the three stations, the Jaccard index was calculated and UPGMA tree was generated using popgen software.

### 2.5. Physiological and Enzymatic assays

Bacterial growth at different temperatures was determined on nutrient broth agar. Plates inoculated and incubated at the following temperatures: 40, 45, 50 and 55 °C. Tolerance to sodium chloride (NaCl) was assessed by determining the growth on nutrient broth agar medium supplemented with 0–12% (w⁄v) NaCl after 7 days incubation at 28 °C.

Amylolytic activity of the cultures was screened using starch agar medium (Merck) [32], followed by incubation at 28 °C for 48 hours. The plates were flooded with 0.3% I_2_–0.6% KI solution. A clear zone around the bacterial growth indicates the hydrolysis of starch. To observe protease production, bacterial cultures were screened in skim milk agar containing 10% skim milk and 2% agar. Clear halos were observed around the bacterial growth after 7 days express protease activity [33] . Lipase production was determined qualitatively on plates by following the method described by Jette and Ziomek [34]. The strains were inoculated on nutrient agar plates containing olive oil (2.5%), Rhodamine (4 mg.L^−1^). The plates were incubated at 28 °C for 48 hours. The orange color under UV is used to identify the lipase producing strains. The presence of Carboxy methyl cellulose activity on plates was determined using a medium containing (g.L^−1^): CMC 10, (NH_4_)_2_ SO_4_ 1.4, K_2_HPO_4_ 2 and MgSO_4_, 7H_2_O 0.02%, nutrient solution 1 (g.L^−1^) (FeSO_4_, 7H_2_O 5 mg.L^−1^, MnSO_4_, H_2_O 1.6 mg.L^−1^, ZnSO_4_, 7H_2_O 1.4 mg.L^−1^, CaCl_2_ 2 mg.L^−1^), agar 20 g.L^−1^. Plates were incubated for 72 hours at 28 °C in the dark. Cellulase activity is indicated by formation of a cleared zone after staining with aqueous Congo red (1 mg.mL^−1^) for 15 min and incubation in 1 mol.L^−1^ NaCl for 15 min [35].

## 3. Results

### 3.1. Physico-chemical characteristics of samples and climatic parameters

**Figure 1.**
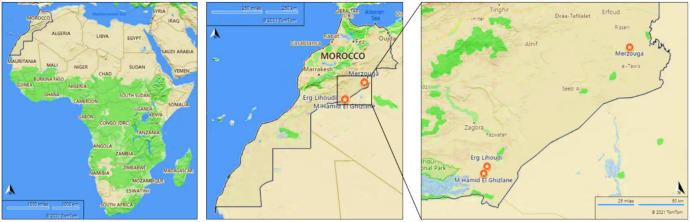
Geographic locations of sampling sites and samples.

**Figure 2.**
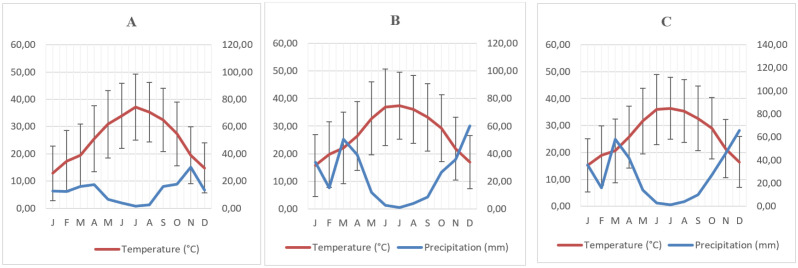
Ombrothermic characteristics of studied regions. (A) Merzouga; (B) Mhamid Elghizlane; (C) Erg lihoudi.

Several physico-chemical parameters were measured from the three samples as shown in Figure 1, the results are shown in Table 1. The Moroccan desert's soil is sandy with fine sand particles as the major fraction, slightly alkaline (pH 8.5), nutrient poor with 0.17% organic content. In the three desert soils, CaCO_3_ and electric conductivity values were between 4.86% and 17.98% and 0.92 mmhos.cm^−1^ and 2.76 mmhos.cm^−1^, respectively. Potassium concentration ranged from 102 to 118 ppm with the highest concentration in Erg lihoudi soil and the lowest concentration in Merzouga soil. Erg lihoudi has significantly higher levels of phosphate and ammonia than all other sites. Climatic parameters, show the monthly average of precipitation and temperature at Merzouga (A), Erg lihoudi (B) and Mhamid Elghizlane (C) sites. The monthly average of precipitation and temperature represents a clear sinusoidal per year at all sites. Annual precipitation was 134 mm, 233 mm and 268 mm at Merzouga, Erg lihoudi and Mhamid Elghizlane, respectively. The monthly average of temperature exhibited large variability. The maximum temperature was 46 °C, 51 °C and 49 °C in June and the minimum temperature was 3 °C, 4 °C and 5 °C in January at the Merzouga, Mhamid Elghizlane and Erg lihoudi, respectively Figure 2.

### 3.2. Bacterial composition and structuration in desert samples

**Figure 3.**
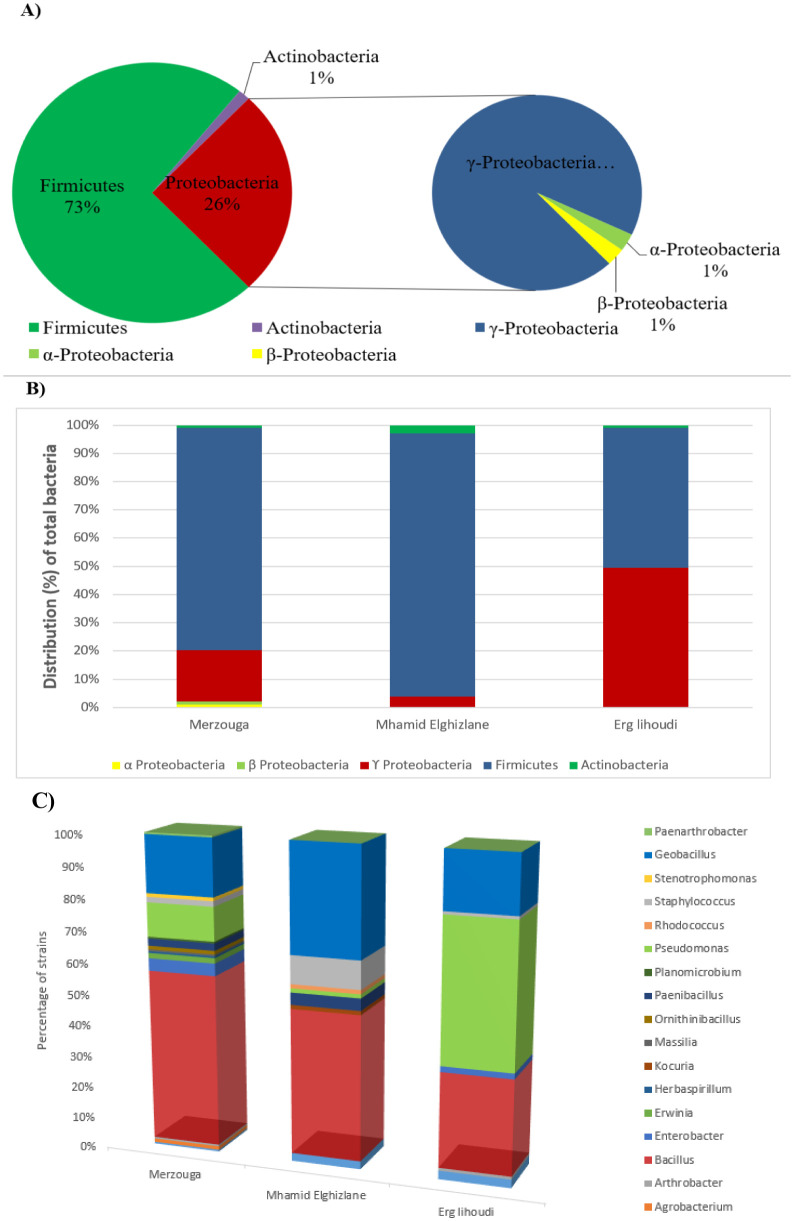
(A) Bacterial composition of the desert samples at the phylum level; (B) distribution of total bacteria in three sampling sites; (C) Bacterial composition of the desert samples at the genera level.

According to the 16S rRNA gene sequences analysis, all the clustered strains revealed an interval of similarity of 99–100%, in the sequences within the GenBank. The phylogenetic tree of the 29 bacterial species identified was constructed. Their affiliations analysis of the 16S rRNA gene sequences revealed that 73.35% of bacterial collection was gram-positive and 26.65% was gram-negative bacteria. Most of these species are Firmicutes species. Interestingly, a greater proportion of Proteobacteria was observed in Erg lihoudi. Overall, 1%, 26% and 73% isolates belong, respectively, to three phyla namely Actinobacteria, Proteobacteria and Firmicutes. The genus *Bacillus* was predominant in Merzouga and Mhamid Elghizlane representing 55% and 46%, respectively, while the genus *Pseudomonas* was the most preeminent genera in Erg lihoudi representing 45% within the region isolates followed by 30% of *Bacillus*. (Figure 3).

Phylogenetic tree of 16S rRNA gene sequences of representative isolates from Moroccan sahara desert and reference sequences generated from GenBank 16S database. 16S rDNA gene sequences were aligned using ClustalW. A Neighbor-joining method was used to build the tree with 1000 bootstraps using MEGA program version X. The GenBank accession No. of the 16SrDNA gene sequences used for phylogenetic tree analysis are indicated at the end of each branch (given the MT Numbers). The *Drosophila montana* (GenBank accession number AF 508191) is used as outgroup.

**Figure 4.**
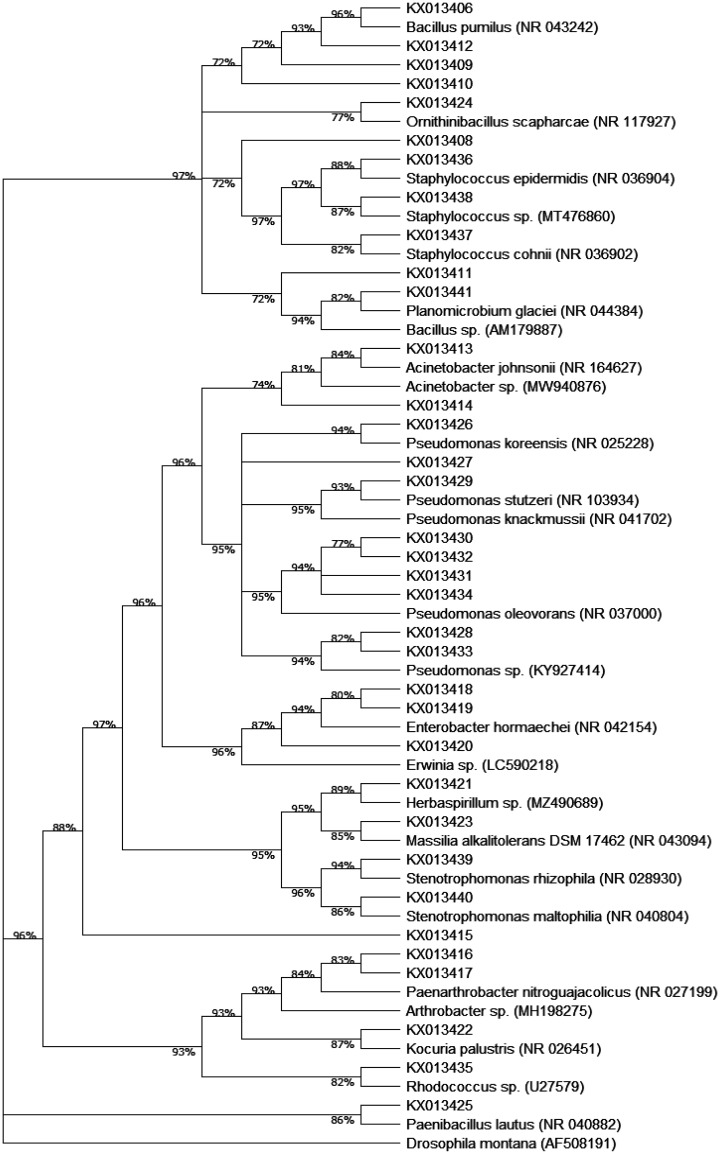
Phylogenetic tree. *Note: Phylogenetic tree of 16S rRNA gene sequences of representative isolates from Moroccan sahara desert and reference sequences generated from GenBank 16S database. 16S rDNA gene sequences were aligned using ClustalW. A Neighbor-joining method was used to build the tree with 1000 bootstraps using MEGA program version X. The GenBank accession No. of the 16SrDNA gene sequences used for phylogenetic tree analysis are indicated at the end of each branch (given the MT Numbers). The Drosophila montana (GenBank accession number AF 508191) is used as outgroup.

Moreover, endophytic population were found in Merzouga such as *Herbaspirllium* sp, *Massilia alkalitolerans* and *Erwinia* sp. Alignment analysis shows different gene sequences among *Bacillus sp* and *Pseudomonas sp*., which need further genetic characterization and validation to identify their taxonomic species. Under phylogenetic analysis, thirteen strains belong to the phylum Firmicutes, which are further distributed into four families: *Bacillaceae*, *Panenibacillaceae*, *Planococcaceae* and *Staphylococcaceae*. Among Firmicutes, six genera represent the phylum whitch are *Bacillus*, *Geobacillus, Ornithinibacillus*, *Paenibacillus*, *Planomicrobium* and *Staphylococcus*. Four strains are affiliated to the phylum *Actinobacteria*, represented by four genera of *Arthrobacter*, *Kocuria*, *Paenarthrobacter* and *Rhodococcus*. Ninety-nine strains belong to the phylum Proteobacteria and distributed into three classes: α-Proteobacteria with *Agrobacterium tumifaciens*; β-Proteobacteria with two strains *Herbaspirillum* sp. and *Massilia alkalitolerans;* and γ-Proteobacteria with twenty-eight strains of five genera. The latter are *Acinetobacter* sp., *Enterobacter* sp., *Erwinia* sp.*, Pseudomonas* sp., *Stenotrophomonas* sp. (Figure 4).

**Figure 5.**
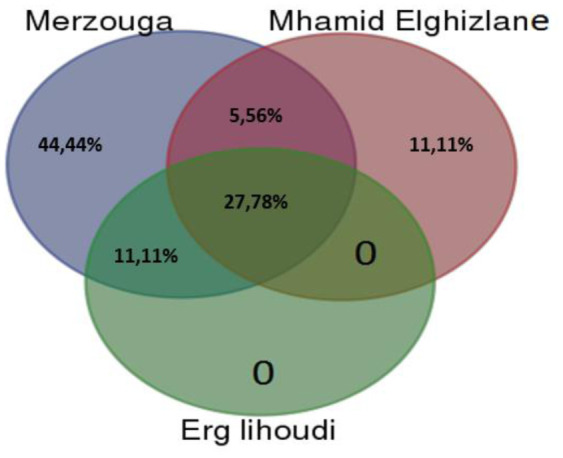
Venn's diagram of the bacterial groups of the desert samples representing the distribution of the percentage of genera. *Note: Venn's diagram represents the percentage of shared and exclusive genera in the three regions.

Overall, *Bacillus, Geobacillus* and *Pseudomonas* are the most frequently recovered genera. All sites have four genera in common including *Bacillus* sp, *Geobacillus* sp., *Pseudomonas* sp., and *Staphylococcus* sp., Analysis of results led to the identification of specific bacterial niche genera at two sites Merzouga and Mhamid Elghizlane. These specific bacterial niches were represented by *Agrobacterium, Erwinia, Herbaspirillum, Massilia, Ornithinibacillus, Planomicrobium, Paenarthrobacter, Stenotrophomonas,* in Merzouga and *Kocuria* and *Rhodococcus* in Mhamid Elghizlane. No special bacterial niche was found in Erg lihoudi station (Figure 5). The results of Simpson's index and Evenness are in agreement with each other representing the highest value in Erg lihoudi and the lowest in both Merzouga and Mhamid Elghizlane (Table 2).

**Table 1.** Physico-chemical properties of samples and sampling sites.

Sampling sites	Clay (%)	Limon fin (%)	Limon coarse (%)	Limon total (%)	Fine sand (%)	Coarse sand (%)	Sable total (%)	CaCO_3_ total (%)	pH	Electric conductivity (mmhos.cm^−1^)	Organic content (%)	NH_4_-N (ppm)	P Olsen (ppm)	K+ (ppm)
Merzouga	3.41	0.54	0.68	1.22	58.85	36.52	95.37	4.86	8.53	0.92	0.18	10.92	7.85	102
Erg lihoudi	3.33	0.23	1.60	1.83	73.62	21.22	94.84	12.27	8.5	2.58	0.16	12.04	11.68	118
Mhamid Elghizlane	0.78	1.26	0.40	1.66	73.68	23.88	97.56	17.98	8.5	2.76	0.17	6.58	7.85	112

**Table 2.** Diversity indices for the isolates from different sites of Moroccan desert.

Locations	Total abundance*	Species Richness	Simpson's (D)	Evenness (EV)
Merzouga	176	25	0.47	0.41
Erg lihoudi	111	9	0.65	0.61
Mhamid Elghizlane	78	9	0.34	0.39

*Note: Total abundance is equal to number of isolates according to16S rRNA data.

**Table 3.** Jaccard similarity index of the desert sand samples.

Locations	Merzouga	Mhamid Elghizlane	Erg lihoudi
Merzouga	1		
Mhamid Elghizlane	0.69	1	
Erg lihoudi	0.57	0.56	1

Similar relationships among the samples are observed in Jaccard index (Table 3).

**Figure 6.**
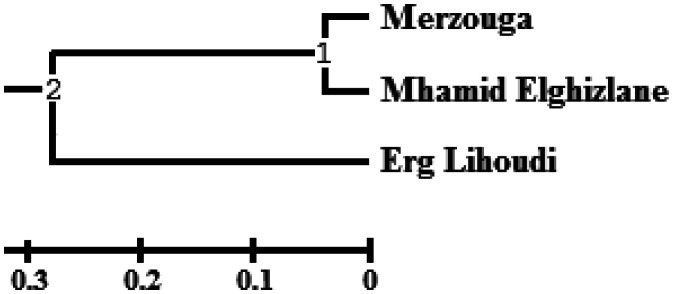
UPGMA tree of the bacterial population of the desert samples. *Note: The tree was generated with popgen. The distance for each samples are indicated by the position of the node between them, according to the Jaccard similarity indices.

The most closely related populations are Merzouga and Mhamid Elghizlane samples UPGMA tree is generated in order to graphically reveal the relationships between these three samples (Figure 6).

Bacterial populations Merzouga and Mhamid Elghizlane samples were found similar and differ from Erg lihoudi sample.

### 3.3. Enzymatic and physiological traits

**Figure 7.**
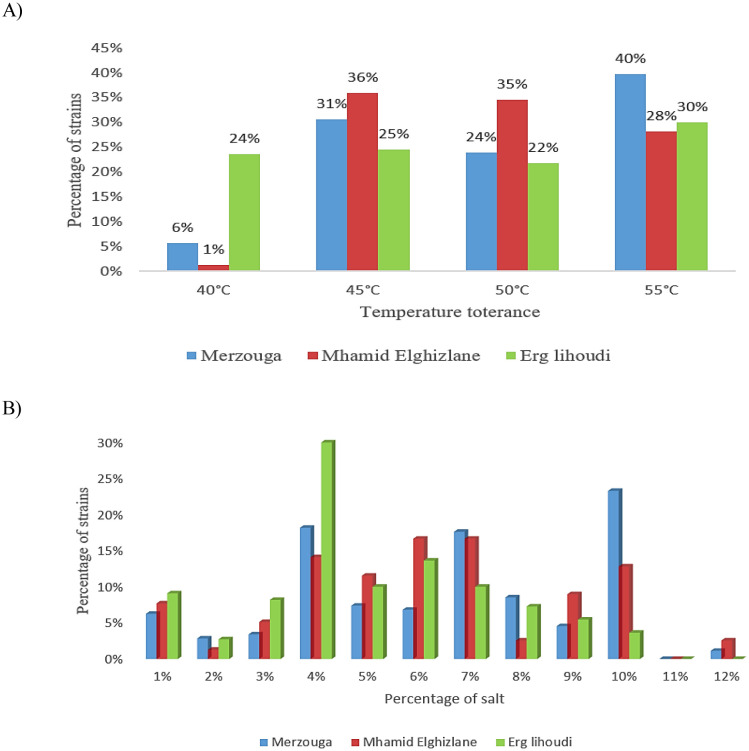
Distribution of bacteria according to temperature (A) and salinity (B) tolerance abilities in different soil samples.

**Figure 8.**
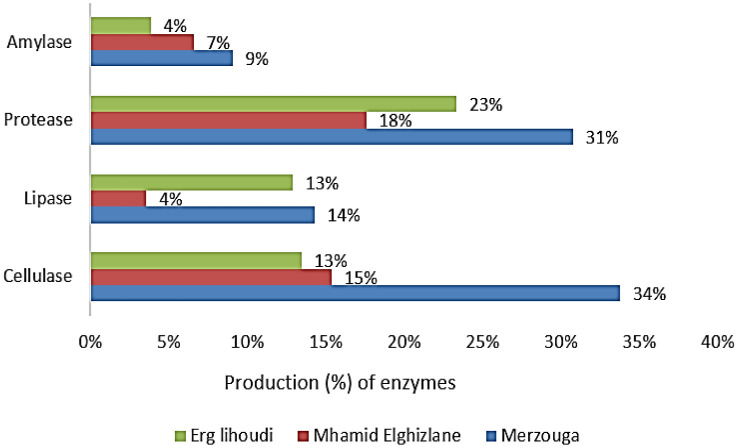
Percentages of enzymes production in each region.

Bacterial strains were screened for thermo-tolerant bacteria at different temperatures 40, 45, 50 and 55 °C. All the strains were able to survive under a temperature of 40 °C. Moreover, rich groups of thermotolerant strains were found representing 40%, 28% and 3% in a temperature of 55 °C in Merzouga, Mhamid Elghizlane and Erg lihoudi, respectively (Figure 7A). Merzouga harbor the highest percentage of halotolerant strains representing 23% at 10% of salt followed by Mhamid Elghizlane which the isolates show their highest tolerance (17%) at 7% Meanwhile Erg lihoudi represent 30% of halotolerant bacteria at 4%. The percentage of halotorant strains decreased while salt concentration increased in the medium (Figure 7B). In the entire collection, 38 strains from two genera, *Bacillus* sp. and *Geobacillus* sp., tolerate to a temperature of 55 °C and 10% of salt, simultaneously, while producing at least two enzymes. The collection of strains were examined for their ability to secrete hydrolytic enzymes. Among all the collection isolates, the distribution ratio of hot active enzyme activities by protease and cellulase producing strains were high (72% and 62%, respectively) compared to Lipase and Amylase (31% and 20%, respectively) in the three regions (Figure 8).

Overall, three strains from two different genus *Pseudomonas* sp. (MDMC 118) and *Geobacillus stearothermophilus* (MDMC153 and MDMC159), from both Mhamid Elghizlane and Erg lihoudi were found able to produce all the studied enzymes.

## 4. Discussion

In the present investigation, a total number of 364 cultivable bacteria were isolated and identified from three different regions in Moroccan Sahara desert. This study revealed that Firmicutes, followed by Proteobacteria, are the most dominant phylum in Moroccan desert soils. These divisions have been observed in different hot deserts of the Tanami Desert, the Eastern Desert, and the Saudi Arabia Desert [36], While Actinobacteria phyla was less prevalent Contrary to other studies conducted in similar locations in the Atacama Desert, Namib Desert, and Thar Desert [37]–[40]. These findings indicate that these phyla have adapted effectively to the scorching desert environment. Their existence could be explained by a variety of processes of desiccation resistance, as extensively described by Heulin et al. [41] including sporulation, hydrobiosis, and encystment, or yet unravelled mechanisms. In contrast, among identified bacteria, the prominent genera isolated are *Bacillus, Geobacillus* and *Pseudomonas*, which is not surprising, *Bacillus, Geobacillus* and *Pseudomonas* genera are ubiquitous in nature and were previously reported in all niches in the environment [42]–[44]. However, 236 isolates remained unidentified to the species level, most of them were assigned to *Bacillus* sp. with 164 isolates and to *Pseudomonas* sp. with 50 isolates. This could be an indication for the presence of potential new interesting species. These isolates need expanding genes analysis including additional metabolic genes [45]. It should be noted that phylogenetic analysis of nucleotide sequences obtained from some strains of the same species revealed 100% similarity, while their colonies morphology and physiological behavior differed significantly in terms of temperature and salinity tolerance, as well as hydrolytic enzyme production. This demonstrates, as Belov et al. [46] confirmed, that these strains are intrapopulation variation. Among firmicutes phylum, the isolation of *Arthrobacter* genus related strains from hot deserts has already been reported in the literature [47] even though it is more likely to be isolated from cold desert [48],[49]. Less commonly genera like *Planomicrobium, Paenibacillus* and *Staphylococcus*, *Geobacillus* have been also reported in several deserts [50]–[52]. While, to our knowledge this is the first report for presence of *Ornithinibacillus scapharcae* in hot desert. This bacteria is usually found in salt lakes [53] which may explain the strains high salt tolerance potential. Contrary to our results, members of proteobacteria have been revealed predominant in several hot deserts, *pseudomonas* in eastern Utah (USA) [54], *Acinetobacter* in Asian deserts such as Gobi (Mongolia) desert and Taklamaken (China) desert [55], *Enterobacter* in Saudi Arabia desert [56]. Representatives of this phyla were also found in different deserts, *Stenotrophomonas* in north Sinai deserts, Egypt [57], *Massilia* from sahara and Gibson deserts, Australia [46]. Noteworthy is the revelation of endophytic species like *Erwinia* sp., *Herbaspirillum* sp. and *Agrobacterium* sp., in complete unvegetated site in Merzouga, may be related to exposure to past vegetation history or by sandstorms in winter and the permanent movement of sand particles carried by the wind and carrying microorganisms clinging to them [58]–[60], since nearby zones contain major vegetation [61]. Minor components of the Actinobacteria phyla's *Arthrobacter, Paenarthrobacter, Kocuria*, and *Rhodococcus* genera were also detected in other hot deserts [16],[62]–[65]. In line with other studies [46], desert ecosystems may harbor additional pathogenic bacteria, as several pathogenic bacteria, including *Erwinia* sp., *Pseudomonas stutzeri*, *and Staphylococcus epidermidis*, were found in three of the stations studied. Furthermore, Invasion of pathogens into desert soil could also be a key element in decreasing microbial diversity [66].

Major physical and chemical aspects affect microbial populations in soils distribution [67]. Interestedly, no significate correlation between the physico-chimic data and bacterial diversity in three station was proven. On the other hand, Erg lihoudi and Mhamid Elghizlane are geographically closer to each other and far away from Merzouga. The two later regions are dominated by Firmicutes and have more similarities in bacterial abundance, with a slight difference in the community structure, than Erg lihoudi which is dominated by Proteobacteria. Based on Venn diagram, biodiversity indices of communities' similarity (Shannon, Evenness and jaccard index). According to jaccard similarity index and related UPGMA tree, in which express the degree of ecological resemblance concerning species composition between the three regions, bacterial abundance in Merzouga and Mhamid Elghizlane stations have shown to be more similar compared to Erg lihoudi. These findings show that abiotic soil characteristics are less involved in the distribution of the microbial communities, as well as regional characteristics. Microbial variability is determined by sampling location, in agreement with other studies [68], since establishing the mains factors controlling diversity remains difficult, microniche variability is given a role in guiding such diversity [69]. In contrast to what generally assumed that the distribution of desert microbes depend essentially on environmental factors [70].

The soil temperature in Moroccan Sahara are subject to day-night variations. It spans from a low level of 2 °C to high level of 50 °C, with poor rainfall values, across the year. Thus, 34% of the bacterial population in the Moroccan Desert tolerates high temperature reaching 55 °C. Based on these data, it is possible to presume that there is a high proportion of strains with thermotolerant and halotolerant properties in the studied soil. As expected, the analysis of the isolates resistance to temperature revealed that more than 59% of the isolates have thermotolerant properties, the highest levels of resistance to temperature were found mainly *Bacillus* genera. Thermotolerant isolates pertaining to *Pseudomonas, Staphylococcus, Paenibacillus, Acinetobacter, Enterobacter, Arthrobacter*, *Geobacillus*, *Ornithinibacillus* and *Planomicrobium* were also found. Representatives of *Bacillus, Paenibacillus and Enterobacter* genera were reported in previous study [56],[71]–[73]. Meanwhile, no previously published reports on thermotolerant behavior of any representatives *Ornithinibacillus* and *Planomicrobium* were found. Furthermore, the finding of the specie *Planomicrobium glaciei* in sahara desert, discovered first in glacier then from desert environments and more often isolated from volcano mud [74],[75] lead us to believe that, in response to extreme conditions prevailing in both cold and hot deserts, the intracellular mechanisms determining the resistance and survivability are adaptable. These results are consistent with the data obtained previously in the other regions of Sahara and Gibson deserts [76]. High tolerance to salt (8%–12%) was found in 80 isolates belonging to the genera B*acillus*, S*taphylococcus* and E*nterobacter*, various forms of halotolerant bacteria had previously been isolated in deserted areas [77]–[79]. As shown, multiple osmotic tolerance is found in *Bacillus, Geobacillus* and *Staphylococcus* genera. In terms of enzyme activity levels, industrial enzymes are running out in tough physicochemical conditions. On the other hand, based on bacteria survival ability under multiple extreme environmental conditions, osmotolerant bacteria have been reported to produce high and stable enzymes [80],[81]. Hence, they could therefore provide potential answers to variety of industrial challenges [82],[83]. Among thermo-halotolerant bacteria recovered, *Geobacillus stearothermophilus* was found able to produce 4 enzymes, cellulase, protease, amylase and lipase [84],[85], Meanwhile no data on cellulase and protease produced by *Geobacillus stearothermophilus* was found. Furthermore, 13 strains all of them from *Bacillus* are capable to produce 3 enzymes, which is well documented [86]–[88]. Additionally, the majority for the isolates represented at least two hydrolytic activities. For instance, the obtained results represent a preliminary step to identify novel valuable enzymes. Hence, this study provides a rich repertoire, from halo-tolerant or thermo-tolerant bacteria producing different hydrolytic enzymes, for further biotechnological concerns.

## 5. Conclusion

To sum up, the current study has shown that Moroccan Sahara Desert has a rich microbial diversity, dominated by *Bacillus*, *Geobacillus* and *Pseudomonas* genera. Recently, high Throughput sequencing techniques were developed [89],[90] which would help to understand the ecological significance of bacterial diversity in the Moroccan Sahara. Likewise, our study provides a collection of 364 isolates among them bacteria capable of producing hot active hydrolytic enzymes of industrial significance. The collection contains a major part of bacteria proven to be halo-tolerant, thermo-tolerant and represents a resource for producing industrial enzymes. The combination of the latter two characteristics reveal interesting candidates for industrial uses. Furthermore, advanced studies will be focalized on hall genome sequencing for each of the candidates.
